# Torpedo Maculopathy

**DOI:** 10.18502/jovr.v15i1.5960

**Published:** 2020-02-02

**Authors:** Vishal Raval, Srinivas Rao, Priyanka Sudana, Taraprasad Das

**Affiliations:** ^1^L V Prasad Eye Institute, KVC Campus, Tadigadapa, Vijayawada, Andhra Pradesh, India

##  PRESENTATION

**Figure 1:**
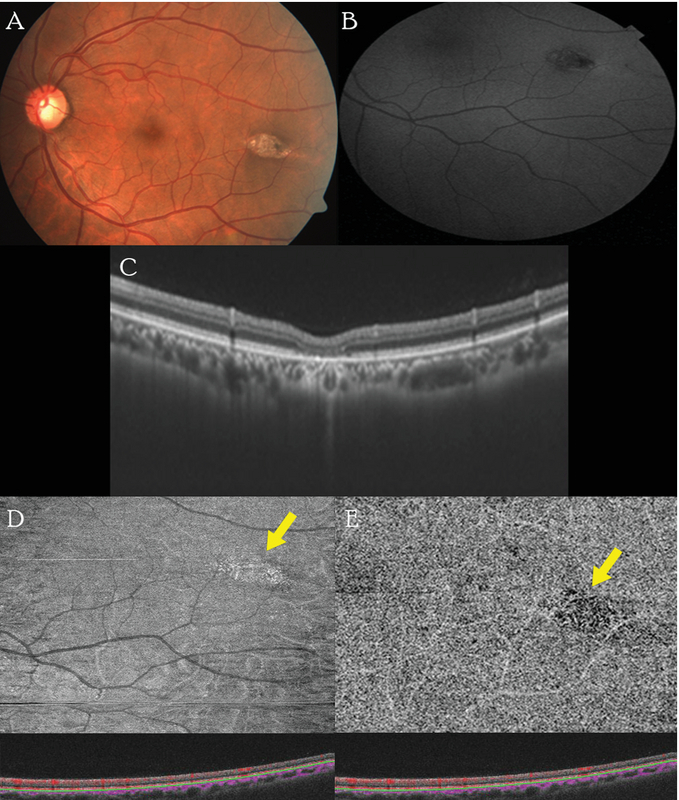
(A) Color photograph of the left eye showing a hypopigmented torpedo-shaped lesion with a hyperpigmented tail. (B) FAF showing hypoautofluorescence with a border of hyper autofluorescence at the tail end. (C) OCT of the lesion showing disruption of the IS-OS junction and atrophy of the outer nuclear layers and increased choroidal reflectance. (D) Infrared reflectance imaging of the choroidal layer showing heterogenous reflectivity with hyperreflectivity at the tail end (yellow arrow). (E) OCT-A of the choriocapillaris layer showing diffuse attenuation of flow signals (yellow arrow).

An asymptomatic 50-year-old male was referred to our institute for examination of a retinal lesion observed temporal to the macula in the left eye. A complete ophthalmic examination was performed with multimodal imaging techniques including fundus photography, fundus autofluorescence (FAF), optical coherence tomography (Topcon Triton Swept-source OCT; Topcon Corporation, Tokyo, Japan), en face OCT, and OCT angiography (OCT-A). His best corrected visual acuity (BCVA) was 20/20, N6 in both eyes. Fundus examination of the left eye revealed a well-defined bullet- or torpedo-shaped lesion observed 2 disc diameter (DD) temporal to the fovea [Figure 1(A)]. The lesion was hypopigmented in nature with a small area of hyperpigmentation at the tail end. FAF showed hypoautofluorescence with a ring of hyperautofluorescence at the tail end of the lesion [Figure 1(B)]. OCT showed normal inner retinal layers, attenuation of the inner segment–outer segment zone with atrophy of the outer nuclear layer, mild outer retinal cavitation, and increased choroidal reflectance [Figure 1(C)]. There was no evidence of any subretinal cleft. Infrared reflectance OCT imaging focused on the choriocapillaris layer revealed a heterogenous area of reflectivity corresponding to the extent of the lesion, with surrounding hyperreflectivity at the tail [Figure 1(D)]. OCT-A segmentation at the superficial capillary plexus was normal, while the choriocapillaris layer showed diffuse attenuation of flow signals with a convoluted pattern of fine vessels and a few empty spaces between them [Figure 1(E)].

##  DISCUSSION

The first case description of torpedo maculopathy was presented by Roseman and Gass in 1992 as an asymptomatic hypopigmented nevus of the retinal pigment epithelium (RPE).^[1]^

The classic appearance described is a solitary hypopigmented lesion that is bullet- or torpedo-shaped, with a wedge-shaped hyperpigmented tail extending outward and pointing toward the foveola along the horizontal raphe.^[2]^ Although its pathogenesis is still unknown, several hypotheses have been proposed: a developmental defect in the nerve fiber layer along the horizontal raphe, abnormal choroidal or ciliary vasculature development and, finally, a persistent developmental defect in RPE in the fetal temporal bulge.^[2]^

On OCT, two patterns of torpedo maculopathy lesions are seen: type 1, showing attenuation of the outer retinal structures without outer retinal cavitation, and type 2, showing both attenuation of outer retinal structures and outer retinal cavitation.^[3]^ On FAF, it usually shows an area of hypofluorescence corresponding to the atrophic RPE, whereas the border of hyperfluorescence surrounding it represents increased accumulation of lipofuscin.^[4]^ The recent introduction of OCT-A modality has provided a better understanding of pathogenesis related to alterations in the choroidal architecture. On OCT-A, normal architecture of superficial and deep capillary plexus is observed, whereas the choroid layer exhibits a diffuse attenuation of choriocapillaris. Giannakaki-Zimmermann et al^[5]^ reported an average decrease of 26% in the signal of the choriocapillaris on OCT-A in torpedo lesions compared with the surrounding areas. As most of the cases are asymptomatic, and the lesion is congenital and non-progressive, observation is recommended in a majority of cases.^[1]^ Rarely, it might be associated with the formation of choroidal neovascular membrane.^[6]^ In conclusion, in torpedo maculopathy, the characteristic OCT-A finding is vascular alterations in the choriocapillaris layer, which supports the hypothesis that the choroid is the first structure to be involved in this condition. Further studies might be needed to provide deeper insights into the pathophysiology of torpedo maculopathy.

##  Financial Support and Sponsorship

Nil.

##  Conflicts of Interest

There are no conflicts of interest.
